# Curation of causal interactions mediated by genes associated with autism accelerates the understanding of gene-phenotype relationships underlying neurodevelopmental disorders

**DOI:** 10.1038/s41380-023-02317-3

**Published:** 2023-12-15

**Authors:** Marta Iannuccelli, Alessandro Vitriolo, Luana Licata, Prisca Lo Surdo, Silvia Contino, Cristina Cheroni, Daniele Capocefalo, Luisa Castagnoli, Giuseppe Testa, Gianni Cesareni, Livia Perfetto

**Affiliations:** 1 Department of Biology, University of Rome Tor Vergata, Via Della Ricerca Scientifica, 00133 Rome, Italy; 2 Human Technopole, Viale Rita Levi-Montalcini 1, 20157 Milan, Italy; 3 Department of Experimental Oncology, European Institute of Oncology IRCCS, Via Adamello 16, 20139 Milan, Italy; 4 Department of Oncology and Hemato-Oncology, University of Milan, Via Santa Sofia 9, 20122 Milan, Italy; 5 Computational Biology Research Centre, Human Technopole, Viale Rita Levi-Montalcini 1, 20157 Milan, Italy; 6 Department of Biology and Biotechnologies ‘Charles Darwin’, Sapienza University of Rome, Piazzale Aldo Moro 5, 00185 Rome, Italy

**Keywords:** Autism spectrum disorders, Neuroscience, Genetics

## Abstract

Autism spectrum disorder (ASD) comprises a large group of neurodevelopmental conditions featuring, over a wide range of severity and combinations, a core set of manifestations (restricted sociality, stereotyped behavior and language impairment) alongside various comorbidities. Common and rare variants in several hundreds of genes and regulatory regions have been implicated in the molecular pathogenesis of ASD along a range of causation evidence strength. Despite significant progress in elucidating the impact of few paradigmatic individual loci, such sheer complexity in the genetic architecture underlying ASD as a whole has hampered the identification of convergent actionable hubs hypothesized to relay between the vastness of risk alleles and the core phenotypes. In turn this has limited the development of strategies that can revert or ameliorate this condition, calling for a systems-level approach to probe the cross-talk of cooperating genes in terms of causal interaction networks in order to make convergences experimentally tractable and reveal their clinical actionability. As a first step in this direction, we have captured from the scientific literature information on the causal links between the genes whose variants have been associated with ASD and the whole human proteome. This information has been annotated in a computer readable format in the SIGNOR database and is made freely available in the resource website. To link this information to cell functions and phenotypes, we have developed graph algorithms that estimate the functional distance of any protein in the SIGNOR causal interactome to phenotypes and pathways. The main novelty of our approach resides in the possibility to explore the mechanistic links connecting the suggested gene-phenotype relations.

## Introduction

### Vulnerability loci in neurodevelopmental disorders (NDDs)

The identification of vulnerability loci that underlie neuropsychiatric disorders has made considerable progress over the past two decades [1]. On the one hand, this has contributed to the realization of their “daunting polygenicity”, with a large number of susceptibility loci contributing to genetic backgrounds of variable and often shared vulnerability across neuropsychiatric categories [2]. On the other hand, this effort has permitted the identification of high penetrance monogenic variants. Together, these insights have broken down highly prevalent diagnostic categories into gradients of risk loadings or a myriad of bona fide rare conditions. This trend is poised to increase, as suggested by the recent estimate that more than 1000 genes associated to different extents with neuro-developmental disorders have not yet been described [3].

In the case of autism spectrum disorders (ASD), different kinds of studies, from both human cohorts and model organisms, have been providing a massive knowledge basis on the genes associated with this condition [4–10]. Results of GWAS studies and additional data have been combined by the curators of the Simons Foundation Autism Research Initiative (SFARI) database to list several hundred genes implicated in autism susceptibility. The SFARI resource [11] associates to the listed genes a score, ranging from 1 (high confidence) to 3 (only suggestive evidence), reflecting the strength of the evidence linking them to ASD (https://gene.sfari.org/). In addition, the syndromic category (S) includes mutations that are associated with a substantial degree of increased risk but are not required for an ASD diagnosis.

Typically, large gene lists are analyzed using methods such as, for instance, over-representation analysis (ORA) or protein interaction network analysis. These approaches are useful to gain insight into the biological functions associated with the genes in the query list and provide hints about the cellular processes whose disruption may contribute to a phenotype of interest. Briefly, methods based on over-representation analysis rely on pathway annotation [12–15] or ontology vocabularies such as the Gene Ontology [16] to investigate whether a gene list is significantly enriched in genes annotated to any given pathway or function. When applying such approaches to the SFARI gene list, a significant enrichment in genes annotated to synaptic regulation and chromatin remodeling is observed [17].

Network representation of biological complexity and graph theory, on the other hand, are playing an increasingly important role in dealing with the intricacy of human physiology and pathology and in limiting the noise that is inherent in large datasets [18]. Network approaches represent protein relationships as graphs connecting physically interacting proteins and build on the observation that related proteins (e.g., true hits from screening experiments or gene products mutated in the same disease) are more connected in molecular interaction networks than random proteins [18, 19].

ORA and network representations, however, have some limitations. On one hand, ORA suffers from the limited annotation coverage in the reference databases as about 40% of the human proteome is not annotated to any pathway by Reactome and KEGG [13, 14] and, as such, they do not contribute to adding information to this analysis. In addition, pathway annotation is biased by curator decisions on whether to assign a protein to a pathway.

On the other hand, networks based solely on evidence of physical interactions, despite having the strength of high proteome coverage [20, 21], cannot provide information about the effects triggered by environmental cues or by genetic perturbations.

**Fig. 1:**
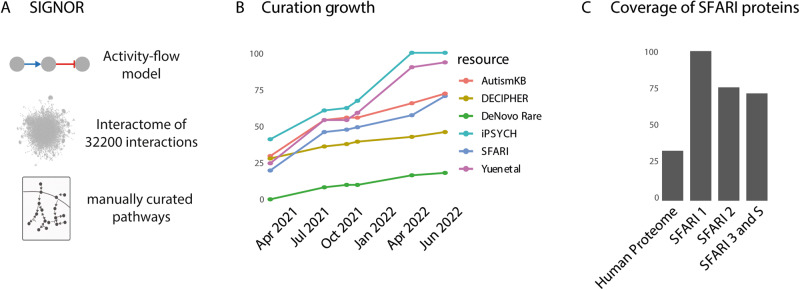
Curation of ASD-related causal relationships and pathways in the SIGNOR resource. **A** Main features of the SIGNOR causal interaction database. Briefly, SIGNOR curators annotate interactions where the activity of one biological entity positively or negatively affects the activity of an interaction partner. The interactions are captured according to an “activity-flow” model, where each interaction is binary, directed and signed (top panel). The interactions are integrated into a global cell network (middle panel). SIGNOR also annotates pathway maps that group set of interactions that modulate the activity of a biological process (bottom panel). **B** Increase in annotation of genes associated to neuropsychiatric diseases by different resources: IPSYCH [28], AutismKB [32], DECIPHER [29], Yuen et al. [30], DeNovo rare [31]. **C** Coverage of SFARI gene lists in SIGNOR. SFARI 1, 2, 3 refer to lists of genes with different supporting evidence for association with autism spectrum disorder as curated by the SFARI resource.

Conversely, networks where the edges are associated with additional causal information such as a direction and a sign are more informative as they allow one to make hypotheses on the causal consequences of the disruption of a protein activity on the function of downstream effectors. In recent years, a number of resources [22, 23] have undertaken an effort to manually capture signaling information from published articles and to represent it in a machine-readable format. The causal information captured by the SIGnaling Network Open Resource (SIGNOR), albeit still incomplete, has the highest coverage of published causal information represented according to the activity flow model [24] (Fig. 1A).

When in early 2021 this resource set out to provide a reference for causal interactions relevant for neuropsychiatric and neurodevelopmental diseases, only ~25% of the genes that had been associated with these disorders were part of the cell interactome in SIGNOR (Fig. 1B).

We report here a curation effort, carried out over the past couple of years, aimed at increasing this coverage. In addition, to showcase the relevance of the curated dataset in dissecting the molecular mechanisms underlying neuropsychiatric diseases, we adapted our recently-developed computational strategy, here dubbed *ProxPath* [25, 26]. *ProxPath* exploits causal information annotated in SIGNOR to extend pathway and phenotype annotation in order to connect a larger fraction of autism-related proteins to a list of cellular pathways and phenotypes. This network-based approach contributes to identifying phenotypes that are “significantly close” to a protein hit list.

## Materials and methods

Methods are fully described in Supplementary Materials.

## Results

### Curation of causal interactions of genes and pathways associated to autism spectrum disorder

Curators of the SIGnaling Network Open Resource (SIGNOR) [22] manually annotate causal interactions according to an “activity-flow” model (protein A up-/down-regulates protein B) [27] (Fig. 1A). The resource captures signaling relationships between a variety of human biological entities, including bio-molecules (proteins, macromolecular complexes, small molecules etc.), stimuli and phenotypes. Interactions in SIGNOR are assigned a significance score, ranging from 0.1 to 1 and form a large and intricate interactome of 9000 entities connected by 34,200 edges (November 2022) (Fig. 1A). SIGNOR causal interactome is a large connected component with few satellite clusters [22]. In parallel, SIGNOR curators also annotate pathways, which are subgraphs of the causal interactome providing a description of how a cell responds to specific environmental cues (Fig. 1A). To date, SIGNOR annotates 114 manually curated pathways.

Here we set out to annotate causal interactions of prioritized ASD-related gene products and cellular pathways (Fig. 1B–D). We took as a reference, for ASD-related genes, the dataset curated by the SFARI initiative [11]. In February 2021 the SFARI gene resource listed 1003 ASD risk genes. At that time, 123/207 score 1 (high confidence), 97/211 score 2 (strong candidate), 210/506 score 3 (suggestive evidence) and 41/79 score S (syndromic) proteins were already included in the SIGNOR cell network.

Since as much as 53% of the genes in the SFARI list were not annotated in SIGNOR, we initially compiled a ranked gene list based on SFARI gene score and prioritized for curation the genes with ascending score (from high to low confidence) that were also listed in other expert-curated resources [28–32].

By this approach, we were able to embed over 300 additional SFARI genes into the SIGNOR causal network and, as a result of this effort, 778 of the 1003 SFARI genes are now annotated in SIGNOR. Of these, the vast majority (770) are part of the large connected cell interaction network, whereas the remaining eight belong to small satellite components that are not connected to the rest of the network (Supplementary Table 1). As shown in Fig. 1C, after this curation effort, 99%, 77% and 71% of the SFARI score 1, 2 and 3 and S proteins, respectively, are now integrated into the cell causal interactome.

### ASD genes form a highly connected cluster in the causal network

**Fig. 2:**
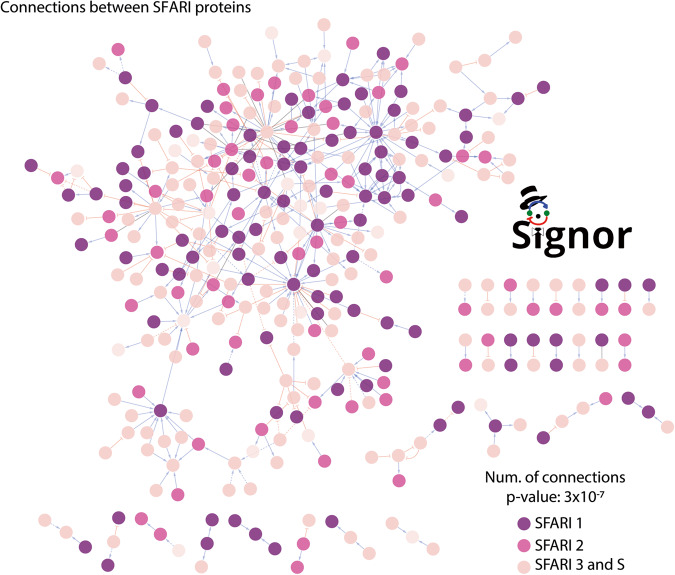
SFARI genes form a connected cluster in SIGNOR. Network of causal interactions directly linking SFARI proteins. The purple color grade of the nodes reflects the SFARI score classification as shown in the legend. Statistical significance of the clustering is measured as a *p* value calculated by comparing the number of direct edges of the displayed network with those of 1000 randomly generated networks linking protein lists of identical size.

In 2017, the group of Barabasi provided evidence that patients affected by the same clinical conditions, despite being characterized by considerable genetic heterogeneity, show a high degree of homogeneity at the pathway level [33]. This is consistent with the notion that the function of genes, found to be mutated in the same disease, often converge onto common signal transduction cascades [34]. We thus tested whether such pathway level convergence of ASD-associated genes is observable in the SIGNOR causal network. To this aim we retrieved from SIGNOR the direct connections between SFARI proteins. As displayed in Fig. 2, SFARI proteins form a large network that is fully connected by 411 directed causal edges, extracted from 285 publications (Fig. 2 and Supplementary Table 2). The *p* value for such a level of connectivity was computed by counting the number of direct connections between SFARI genes in 1000 networks where the connections are randomized, while maintaining node degree and edge direction distribution [35]. The calculated *p* value is in the order of 3*EXP-7 (Fig. 2). KEGG over-representation analysis reveals that this network is enriched in proteins annotated with ontology terms “Long-term potentiation”, “Glutamatergic synapse”, “Dopaminergic synapse” and “Circadian entrainment” (Supplementary Table 3) (see “Methods”).

Next, we aimed to see whether SFARI genes tend to form clusters (i.e., densely connected regions) within this network. To this aim we performed a clustering analysis by employing a Random Walk community detecting algorithm [36]. By this approach we were able to detect four major communities (Supplementary Table 3). KEGG and GO over-representation analysis reveals that these comprise proteins that participate in neuronal development, synaptic processes and neurotransmitter metabolism (Supplementary Table 3).

### Mapping ASD genes to pathways whose perturbation has been implicated in neurological disorders

**Fig. 3:**
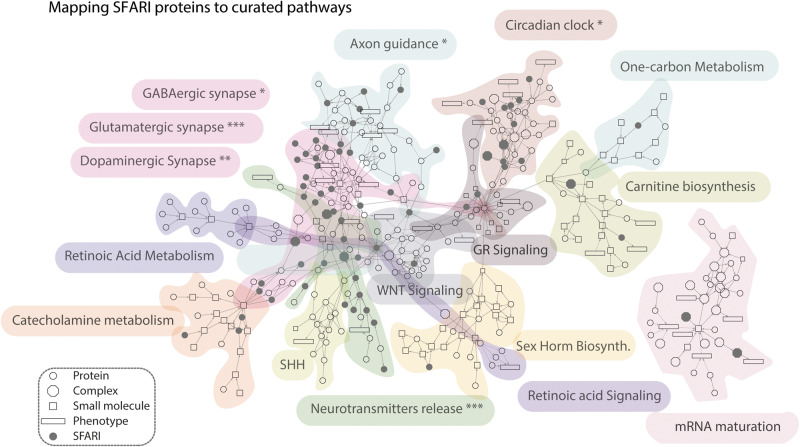
Mapping SFARI prioritized genes onto pathways of neurological relevance. The scaffold network is obtained by merging pathway maps curated in SIGNOR. Nodes are biological entities: circles correspond to proteins, rectangles to phenotypes, squares to small molecules, octagons to protein complexes. Black nodes indicate SFARI proteins or complexes containing SFARI proteins. Significance of enrichment of SFARI genes in the considered pathways was evaluated by a Fisher’s exact test using as background all the proteins in SIGNOR (Bonferroni-corrected *p* value: *<0.05, **<0.01, ***<0.001). Mapping SFARI genes on such a graph (dark gray nodes) permits focusing on the pathways that are significantly enriched in the SFARI gene list.

In parallel to the gene and phenotype annotation work, we have also curated a list of 16 pathways that have been reported to be linked to ASD (the complete list of pathway and pathway members is reported in Supplementary Table 4). They include signal transduction cascades governing neuron development and differentiation, synaptic assembly and transmission. In addition, we have also curated pathways that were found perturbed in ASD patients (e.g., Sex Hormone Biosynthesis [37] or WNT [38]), or biological processes that emerged from the analysis of ASD-related genes (e.g., mRNA maturation) [39]. The curated pathways, with the exception of the “mRNA maturation” pathway, form a single connected cluster (Fig. 3).

As shown in Fig. 3, SFARI proteins (black circles) preferentially participate (*p* value < 0.05) in pathways involved in neurotransmitter release or synaptic transmission. Interestingly, the observation of a significant SFARI-protein enrichment in the circadian clock pathway provides support to the suggestion that dysregulation of the circadian rhythms plays a role in autism spectrum disorder [40]. While this type of analysis is reminiscent of a conventional pathway enrichment analysis, it provides additional crucial information. The mapping of disease genes to pathways embedded in a causal network enables in fact the following: (1) inspect pathway cross-talk, (2) formulate hypotheses on the mechanisms that are disrupted in the diseases and (3) provide suggestions on how to revert the disease phenotype by network intervention. The details of the network in Fig. 3 can be inspected at https://www.ndexbio.org/viewer/networks/fbc6ec1f-fe96-11ec-ac45-0ac135e8bacf .

### Pathway and phenotype annotation extension

**Fig. 4:**
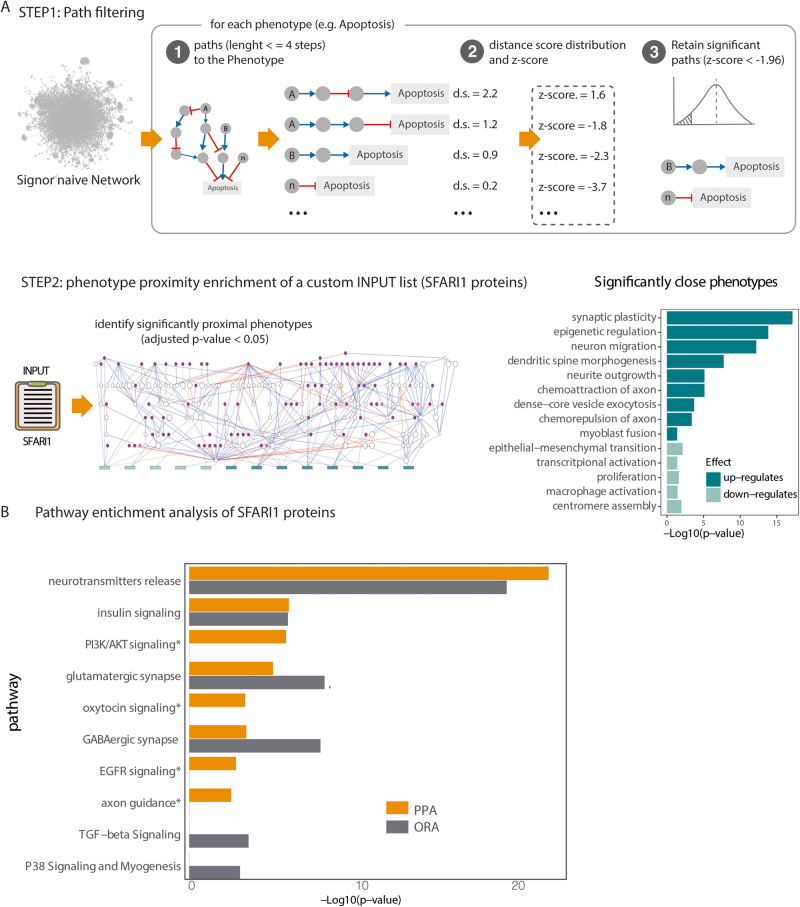
Estimating the regulatory impact of SFARI (score 1, SFARI1) proteins on phenotypes and pathways annotated in SIGNOR. **A** Step1: the SIGNOR causal network is filtered to retain only significantly short paths impacting on phenotypes: Step1.1: for each protein-phenotype pair we calculate all the weighted distances in paths shorter than four steps; Step1.2: for each phenotype we plot a distance distribution and for each path we calculate the *Z*-score; Step1.3: proteins with a *Z*-score lower than −1.96 (*p* value < 0.05) are considered to be likely to modulate phenotype expression. Step2: significant paths extracted from Step1 are used to infer the phenotypes that are significantly close to a custom list of proteins (e.g., SFARI1 proteins), via *t*-test statistical analysis. The results of the enrichment can be visualized as networks of causal interactions (left panel) or as bar diagram (right panel). **B** Comparison of enrichment analysis by over representation (ORA) and pathway proximity (PPA) considering as input the SFARI1 list of genes.

The global causal cell interactome curated in SIGNOR allows to connect each pair of biological entities via weighted and directed graph-paths (Fig. 4). The causal network also links cell pathways and phenotypes to proteins that have the potential to modify their activities. Embedding pathways and phenotypes into the cell causal network allows one to walk along the directional edges of the network and to estimate a causal distance between a protein and a phenotype or a pathway. To support this type of analysis we recently implemented *ProxPath*, an algorithm that, given a set of proteins, estimates its regulatory impact over phenotypes and pathways annotated in SIGNOR [26] (Fig. 4B). *ProxPath* identifies short causal paths linking two graph nodes and estimates their *functional distance*. The approach considers the “trust score” of each graph edge, thus allowing to define quantitatively if and how the activity of a protein has the potential to modulate the activity of a phenotype or a pathway. In the SIGNOR-network pathways are collections of causally connected nodes while phenotypes are individual nodes. Approximately 200 phenotypes and 114 pathways are presently embedded into the SIGNOR cell causal network.

We here describe two applications of *ProxPath*: the first measures the functional distance of an input gene list from individual target nodes (e.g., phenotypes), whereas the second computes the regulatory distance of an input list of genes to lists of target nodes (e.g., proteins that belong to a pathway).

The distribution of functional distances of proteins from a phenotype (or a pathway) depends on how central the phenotype or the pathway is in the causal graph. Thus, it is crucial to first normalize the potential impact of a certain protein on the modulation of the target entity. To this end, for each phenotype, *ProxPath* first plots a distribution curve of the weighted distances of all proteins from the phenotype (see also “Methods” section). The activity of proteins with a distance-distribution *Z*-score smaller than the significance threshold −1.96 (i.e., −2 standard deviations) are considered to be significantly close (small distance) and as having a significant chance of impacting the phenotype or pathway (Fig. 4A, step 1.3). This strategy allows us to extend the association of proteins to phenotypes and pathways in an unbiased manner and to make it quantitative and in that respect independent of curators’ decisions on the proteins to be associated to a pathway or a phenotype. By this approach we label each protein as significantly close to any of the SIGNOR phenotypes depending on whether the path distance has a *Z*-score <−1.96 in the distance distribution curve (Fig. 4A). A similar approach is used to identify proteins that are significantly close to a pathway, as already described [25].

In essence, this approach allows to extend node annotation to nodes that are functionally close and relieves the approach from biases caused by curator decisions, thus enhancing the power of functional enrichment analysis. In this perspective *ProxPath* is similar, in scope, to existing network diffusion approaches [41]. To perform a direct comparison, we applied a network propagation algorithm using the heat diffusion implementation provided by Carlin et al. [42] and compared the results to *ProxPath*. As demonstrated in Supplementary Fig. 1, the two approaches identify similar sets of phenotypes. However, *ProxPath* offers additional layers of information, such as the causal effects (up- or down-regulation) on the target phenotype (Supplementary Fig. 1 and Supplementary Table 5).

In support of this approach we compared the results of pathway enrichment of curator annotated pathways with that obtained after pathway expansion by causal pathway proximity (PPA), obtained by applying *ProxPath*. To this end, as outlined in the “Methods” section, we used the same input (the SFARI 1 gene list), background (the entire human proteome) and multiple-testing correction method. As shown in Fig. 4B, both approaches identified “neurotransmitter release”, “insulin signaling”, “glutamatergic synapse” and “gabaergic synapse” as significantly enriched pathway annotations. However, differences were also observed as pathway proximity analysis revealed in addition the “PI3K/AKT signaling”, “oxytocin signaling”, “EGFR signaling” and “axon guidance” pathways, whose perturbations have already been described in autism spectrum disorders [43–45]. In summary, our PPA approach has recapitulated the results from standard ORA, while partially compensating for the incomplete coverage of resources ORA depends on (Fig. 4B).

We have also asked whether the SFARI1 gene list is significantly enriched for proteins that are functionally close to any of the 200 phenotypes annotated in SIGNOR. To this end we generated 1000 lists of random proteins and computed a *p* value for a random list having a number of proteins, significantly close to each phenotype, which is equal or larger than that observed in the SFARI1 list. Although this strategy has also a certain degree of arbitrariness, it provides an independent estimate of gene-phenotype association. We confirm that the SFARI1 gene list is enriched for genes that have the potential of positively modulating phenotypes related to synapsis assembly and function (Fig. 4A). Furthermore, the approach also revealed an enrichment of genes involved in “epigenetic regulation” and “dense core vesicle exocytosis”, processes which were already associated with autism spectrum disorders [46, 47].

In summary, these analyses show that networks of causal interactions are useful to describe cellular processes or pathways that are associated to a list of gene products and can partially alleviate the lack of coverage in pathway resources. In addition, as the approach identifies the causal interactions linking the query proteins to a phenotype, it makes it possible to draw a graph detailing the molecular steps by which the proteins in the list may impact the phenotypic expression (Fig. 4A, B). The complete network of causal interactions delineating the paths impacting enriched phenotypes can be inspected at https://www.ndexbio.org/viewer/networks/b7d7e952-fe97-11ec-ac45-0ac135e8bacf.

### Integrating poorly characterized proteins into the cell causal network provides information on their functions

Not all proteins in the human proteome are equally well-annotated. The “Illuminating the Druggable Genome” (IDG) project has developed a web-based platform (Pharos) that aggregates functional information captured by over 60 resources [48]. Pharos uses a knowledge-based classification system to rank proteins according to the degree to which they are studied, as evidenced by a variety of features, thus helping to identify less characterized proteins. In total, 5932 understudied proteins whose functions have been poorly, or not at all, characterized are labeled as “understudied” and form the Tdark proteome.

**Fig. 5:**
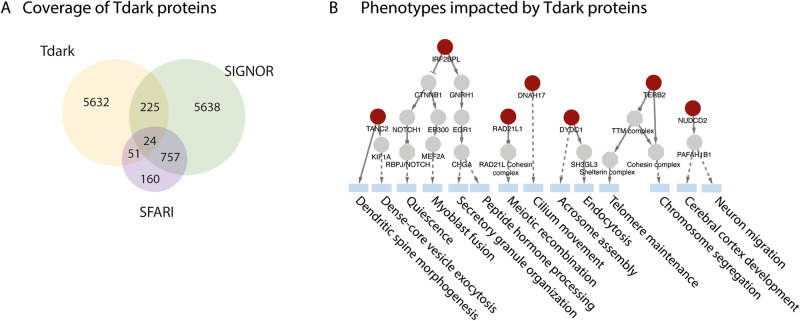
Tdark proteins coverage. **A** Overlap of proteins annotated in three resources: SIGNOR, SFARI and Tdark [48]. **B** Causal paths linking SFARI proteins to phenotypes annotated in SIGNOR. Tdark proteins are in red.

Seventy-five SFARI proteins are part of the Tdark proteome and, as a consequence, hardly any experimental evidence can support generation of hypotheses on the mechanisms underlying their contribution to ASD (Fig. 5A). However, 24 of the 75 SFARI proteins that are classified as Tdark according to Pharos are part of the SIGNOR causal interactome and can link to nodes in the network, including phenotypes. As examples, four causal edges or fewer can link NUDCD2 to *cerebral cortex development*, TANC2 to *dendritic spine morphogenesis* and IRF2BPL to *secretory granules organization*, three phenotypes that have already been implicated in ASD [49–51] (Fig. 5B). These observations point to the potential of a strategy based on linking poorly characterized genes to the cell causal interactome to shed light on their function.

### Adding support to rank genes with suggestive evidence of association to ASD

Over recent years, the SFARI-gene resource has collected genetic evidence from genome-wide association studies to link human genes to autism spectrum disorders (ASD). Close to 1000 genes have been potentially linked to ASD. Thus, it is important to rank them according to the strength of the supporting evidence to prioritize candidate genes for time consuming follow up experiments. SFARI curators have grouped ASD genes into four score categories: 1 to 3, with decreasing levels of supporting evidence, and S (Syndromic genes).

As the large number of genes may cause the inclusion of false positives, especially in the categories with lower experimental support, additional and orthogonal scoring strategies may be useful to further prioritize genes. This is particularly true for those genes that are only included in the lists because of suggestive evidence.

We argue that genes whose activities underlie a given phenotype or whose disruption may contribute to a disorder are likely to be closely connected in a causal network. Thus, we took the list of high confidence SFARI genes (SFARI1) as a proxy of bona fide genes modulating the ASD phenotype. Next, for each gene in the proteome we used the causal distance from the closest gene in the SFARI 1 gene list as an estimate of their potential to be an ASD susceptibility gene. This approach generates a ranked gene list where the genes placed at the top of the list are the ones that receive more support from our network approach to be functionally connected to ASD (Supplementary Table 6).

As a proof of concept, we tested whether independently-defined lists of ASD-associated genes were *de facto* enriching top-ranked genes (those showing higher probability of being functionally connected to SFARI1 proteins). To this aim, we performed Gene Set Enrichment Analysis (GSEA) and demonstrated that both the PCMI-ASD genes [52] and the physical interactors of ASD proteins from Pintacuda et al. [53] are enriched in top-ranked genes (genes close to SFARI1 proteins—our bona fide dataset), whereas a set of randomly selected proteins were not. Moreover, by this comparison we could also show that the level of enrichment of SFARI1, 2 and 3 genes correlate with the confidence score, providing trust in the robustness of the approach (Supplementary Fig. 2).

### Common etiology of neuropsychiatric disorders

**Fig. 6:**
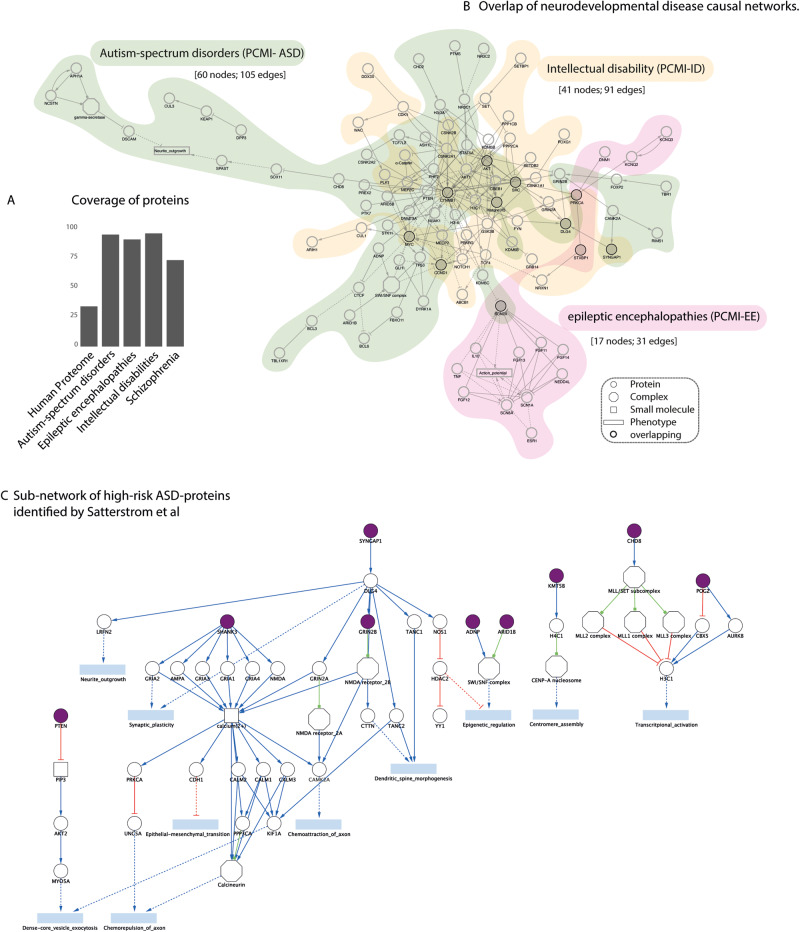
Overlap of neurodevelopmental disease causal networks. **A** Percent coverage of different neuropsychiatric-disease gene lists in SIGNOR. Schizophrenia; autism-spectrum disorders; epileptic encephalopathies; intellectual disabilities refer to the disease gene lists compiled by the Psychiatric Cell Map Initiative [52]. **B** Three disease networks are obtained by querying the SIGNOR resource with the neurodevelopmental disorders proteins annotated by the Psychiatric Cell Map Initiative to Autism spectrum disorders (PCMI-ASD), Intellectual disability (PCMI-ID), Epileptic encephalopathies (PCMI-EE). The three networks are shaded with green, yellow and pink backgrounds respectively. Nodes are biological entities: circles correspond to proteins, rectangles to phenotypes, squares to small molecules, octagons to protein complexes. Proteins that are common to more than one disease map are represented as black-circled nodes. **C** Sub-network of causal interactions extracted from the interactome in Fig. 4A, linking 9 out of 13 high-risk ASD genes (in purple) [6] to deregulated phenotypes (in blue).

We next asked whether the value of our curation effort aimed at the integration of ASD genes into a cell causal network is not limited to the interpretation of the SFARI dataset and could more generally be valuable in neuropsychiatric studies. To this aim we monitored the annotation coverage of independently-defined lists of genes implicated in neuropsychiatric disorders, as reported by the Psychiatric Cell Map Initiative [52] (Fig. 6). The overall goal of this initiative is that of connecting genomic data to functional data (e.g., physical and genetic interactions) and ultimately to the clinic. Here we focus on autism spectrum disorders (ASD), intellectual disability (ID), epilepsy (EP), epileptic encephalopathies (EE) and Schizophrenia (SCZ). To avoid confusion the list of genes associated with ASD by the Psychiatric Cell Map Initiative will be indicated as “PCMI-ASD”.

We observe that our curation project also resulted in a high annotation coverage of these independently-defined gene lists (Fig. 6A). Only 23 out of 47 proteins from PCMI-EE, PCMI-SCZ and PCMI-ID lists are also in the SFARI lists. Nevertheless, their coverage in the SIGNOR interactome is over 80%. Based on this observation, we speculate that the causal interactions annotated in SIGNOR are not limited to the SFARI dataset and to ASD but have also value for a broader range of neurodevelopmental and neuropsychiatric disorders.

To show this point, for each disease, we used the list of genes associated to four neuropsychiatric disorders, as annotated by the PCMI, to query the SIGNOR resource using the “connect + add bridge proteins” search method [54]. This method uses the causal information annotated in SIGNOR to draw networks indirectly connecting query proteins with bridge proteins. The three resulting graphs were displayed in a single layout, forming a connected network (Fig. 6B). No interaction paths could be drawn to link the PCMI-SCZ proteins. The PCMI-ID and PCMI-ASD maps appear to be highly interconnected and share nodes and edges (Supplementary Fig. 3). Several proteins are common to the disease maps of more than one disorder (Fig. 6—gray nodes and Supplementary Fig. 3). These include proteins that take part in the WNT and the Focal Adhesion pathways, suggesting that the deregulation of these biological processes might be implicated in more than one neurodevelopmental disease. These observations support the notion that the three disorders have a partially common molecular etiology.

### Leveraging the SIGNOR causal interactome to identify molecular convergences of ASD forms

We finally asked whether a causal network approach could recapitulate existing knowledge and provide hypothesis-generating observations. The genetic landscape of ASD is highly heterogeneous [55], including highly penetrant variants (both mutations and copy number variations (CNV)), often occurring de novo, and a large number of single nucleotide polymorphisms (SNPs) interacting within individual genetic backgrounds [5, 56] as well as environmental factors [9]. Large GWAS and exome-sequencing studies have highlighted the existence of genes that were found more frequently mutated in a cohort of more than 12 K patient-derived samples [6]. This suggests that these genes play a preeminent role in shaping the condition phenotype. Moreover, thirteen of such genes fall within loci that are more recurrently hit by copy number variations. These thirteen genes are annotated in SIGNOR. Hence, we extracted from the network described in Fig. 4A the subgraphs connecting these genes to significantly close phenotypes via causal relationships. Only 9 of the 13 genes could be connected to phenotypes via causal paths that are significantly short and are represented in the graphs in Fig. 6C. By this approach we could identify two gene groups that recapitulate previous knowledge, a first one impinging on synaptic and neuronal activity and a second bearing on epigenetic regulation and transcriptional control.

Although the two functional gene clusters are separate, the network suggests they might crosstalk via the postsynaptic scaffolding protein DLG4. DLG4, which plays a critical role in synaptogenesis and synaptic plasticity, appears to impact epigenetic regulation by negatively modulating the activity of HDAC2 via nitrosylation by nNOS, in the graph. A second level of crosstalk, revealed by our network, is represented by the chromatin regulators ADNP and POGZ that cooperate in modulating the expression of multiple clusters of synaptic genes, and whose mutation leads to a significant decrease of postsynaptic protein expression and glutamatergic transmission [57, 58].

The cross-talks between the “synaptic” and “epigenetic” axes, as revealed by network analysis of the causal connections between disease genes and phenotypes, could explain the phenotypic similarity of neurodevelopmental disorders caused by germline mutations of regulators of these two axes [17]. The synthetic perturbation of the activity of such regulators [59] could thus translate into similar electrophysiological endophenotypes, because of their functional proximity. However, given the centrality of chromatin remodeling into defining differentiation trajectories, we cannot exclude that this functional proximity could instead translate into differentiation biases and non-cell-autonomous effects that globally result in similar phenotypes. Indeed, recent work has shown that perturbation of 36 high-risk ASD genes in cortical brain organoids converge toward differentiation biases, whereas functional analysis of the top dysregulated genes also refer to cell adhesion and “axogenesis” [60].

## Discussion

Autism spectrum disorder (ASD) is a neurodevelopmental condition, frequently caused by mutations of synaptic and chromatin regulators. The condition is characterized by early onset and results in individually variable socio-cognitive impairments. The past decade has witnessed a major shift in our view of NDD as conditions potentially amenable to pharmacological interventions specifically geared to their causative mechanisms, as exemplified in the paradigmatic cases of Fragile X syndrome [61], Down syndrome [62] and 7q microduplication syndromes (7Dup) [63, 64]. However, the difficulty in translating Fragile x insights from preclinical models to the human setting has also highlighted how key knowledge gaps still hamper such translational pipelines. This becomes all the more relevant if we are to pursue the daunting polygenicity of ASD into rational subsets stratified by convergent and actionable molecular alterations. Toward this long-term goal, here we aimed at providing a resource for the community to streamline the identification of causal interactions between ASD vulnerability genes and hence of the most likely hubs of convergent dysregulation to be prioritized for experimental validation and translational pipelines. Specifically, we report two advances that help to elucidate the molecular mechanisms underlying the involvement of gene variants in disease onset and development. First, expert curation has screened the literature and captured experimental information on the consequences of disrupting disease gene functions on the activity of downstream genes. This information has been integrated into a large cell causal network representing how the activity of gene products crosstalk and impact pathway expressions and phenotype manifestations. Thanks to this curation effort, over 90% of autism-associated gene products are now integrated into the cell interactome and causally connected to the remaining gene products, pathways and phenotypes. The results of this project are now publicly available and can be freely explored by using tools offered by the SIGNOR resource website or downloaded for local analysis, in compliance with the FAIR principles [65]. The cell interactome can be navigated by graph algorithms and the mechanistic steps leading to functional crosstalk of any gene pair can be explored by navigating the network. Although in this project the focus of the curation were genes implicated in ASD, the network that we have assembled also includes many genes involved in other neuropsychiatric conditions and its utility can therefore be extended to all of them.

Second, we have developed graph algorithms that allow us to measure the functional distance of any gene from any pathway or phenotype, by leveraging the features of SIGNOR graph, which is directed and signed, and whose edges are weighted according to estimated supporting evidence.

Given the phenotypic and genetic complexity of autism spectrum disorders and the number of genes that have been found to be associated to these conditions, the literature abounds of suggestions of association of ASD genes to perturbation of practically any cell function [66, 67]. Nevertheless, our approach provides independent evidence of some of these connections and offers the unprecedented opportunity to contextualize their interrelations. Here, we have used the above-mentioned algorithms to show that autism-associated genes are significantly more proximal to pathways and phenotypes involved in functions that underlie brain development. Moreover, our analyses have revealed significant functional connections with a sizable specific portion of cellular pathways, implicated in transcriptional and epigenetic regulation. Here, we claim that our approach not only allows us to connect genes to functions via causal links but it also provides suggestions on the mechanistic steps underlying these connections.

We have demonstrated the value of embedding disease genes into a causal cell interactome in order to formulate hypotheses on the molecular mechanisms leading to phenotypic perturbations caused by gene variants. It needs to be pointed out, however, that the cell causal interactome that is presently covered by the SIGNOR dataset is still incomplete and includes only 33% of the human proteome. As a consequence, some relevant causal links may be still missing and this may somewhat alter the analyses of gene-pathway distance. Furthermore, the cell causal network that we have presented here is obtained by integrating evidence from experiments performed in a variety of cell types, tissues or model systems. Many of these causal relationships may not be relevant for the function of the cell type that is affected during brain development in autism patients. Approaches to exploit single cell RNAseq datasets to develop cell type specific interactomes have been proposed [68] and applications of these strategies to the SIGNOR dataset may help to assemble more biologically and clinically relevant cell interactomes. Nevertheless, our approach, while recapitulating existing knowledge, has revealed gene-phenotype/pathway connections that suggest mechanistic steps underlying such connections.

### Supplementary information

Supplementary methods

Supplementary Table S1

Supplementary Table S2

Supplementary Table S3

Supplementary Table S4

Supplementary Table S5

Supplementary Table S6

## Data Availability

Curated data are available at https://signor.uniroma2.it/downloads.php.

## Code availability

Developed code is available at https://github.com/SaccoPerfettoLab/ProxPath.
