# Correction: Curation of causal interactions mediated by genes associated with autism accelerates the understanding of gene-phenotype relationships underlying neurodevelopmental disorders

**DOI:** 10.1038/s41380-024-02432-9

**Published:** 2024-01-24

**Authors:** Marta Iannuccelli, Alessandro Vitriolo, Luana Licata, Prisca Lo Surdo, Silvia Contino, Cristina Cheroni, Daniele Capocefalo, Luisa Castagnoli, Giuseppe Testa, Gianni Cesareni, Livia Perfetto

**Affiliations:** 1 Department of Biology, University of Rome Tor Vergata, Via Della Ricerca Scientifica, 00133 Rome, Italy; 2 Human Technopole, Viale Rita Levi-Montalcini 1, 20157 Milan, Italy; 3 Department of Experimental Oncology, European Institute of Oncology IRCCS, Via Adamello 16, 20139 Milan, Italy; 4 Department of Oncology and Hemato-Oncology, University of Milan, Via Santa Sofia 9, 20122 Milan, Italy; 5 Computational Biology Research Centre, Human Technopole, Viale Rita Levi-Montalcini 1, 20157 Milan, Italy; 6 Department of Biology and Biotechnologies ‘Charles Darwin’, Sapienza University of Rome, Piazzale Aldo Moro 5, 00185 Rome, Italy

**Keywords:** Autism spectrum disorders, Neuroscience, Genetics

Correction to: 10.1038/s41380-023-02317-3, published online 15 December 2023

In this article, Author’s Contribution statement should have appeared as below.

Current text:

**AUTHOR CONTRIBUTIONS**

Conceptualization: LP, GC and GT; methodology: LP and GC; software: PLS; formal analysis: LP, AV and DC; investigation: LP; resources: GC, GT and LP; data curation: MI, LL, SC, PLS and LC; writing—original draft preparation: LP, GC, GT, MI and AV; writing—review and editing: LP, GC, GT, MI and AV; visualization: LP, PLS and GC; implementation of bioinformatics strategy: LP, PLS and AV; supervision: LP, GC and GT; project administration: GC and LP; funding acquisition: GC, LP and GT. All authors have read and agreed to the published version of the manuscript.

Text with desired changes:

**AUTHOR CONTRIBUTIONS**

Conceptualization: LP, GC and GT; methodology: LP and GC; software: PLS; formal analysis: LP, AV and DC; investigation: LP; resources: GC, GT and LP; data curation: MI, LL, SC, CC, PLS and LC; writing—original draft preparation: LP, GC, GT, MI, CC and AV; writing—review and editing: LP, GC, GT, MI, CC and AV; visualization: LP, PLS and GC; implementation of bioinformatics strategy: LP, PLS, DC and AV; supervision: LP, GC and GT; project administration: GC and LP; funding acquisition: GC, LP and GT. All authors have read and agreed to the published version of the manuscript.

The original article has been corrected.

