# Supplementary material for: Curation of causal interactions mediated by genes associated with autism accelerates the understanding of gene-phenotype relationships underlying neurodevelopmental disorders
Source: Mol Psychiatry. 2023 Dec 15;29(1):186–96. doi: 10.1038/s41380-023-02317-3 (PMC11078740; doi:10.1038/s41380-023-02317-3)
Supplement: Supplementary file 1 — Supplementary methods [file 41380_2023_2317_MOESM1_ESM.pdf]

Supplementary materials

# Curation of causal interactions mediated by genes associated to autism accelerates the understanding of gene-phenotype relationships underlying neurodevelopmental disorders

Marta Iannuccelli[1*], Alessandro Vitriolo[2,3,4,*], Luana Licata[1,5], Lo Surdo Prisca[1,5], Silvia Contino[1], Cristina Cheroni[2,4], Daniele Capocefalo[2,4] , Luisa Castagnoli[1], Giuseppe Testa[2,3,4,#] Gianni Cesareni[1,#], Livia Perfetto[5,6,#]

[1] Department of Biology, University of Rome Tor Vergata, Via della Ricerca Scientifica, 00133, Rome, Italy
[2] Neurogenomics, Human Technopole, Viale Rita Levi-Montalcini 1, 20157, Milan, Italy
[3] Department of Experimental Oncology, European Institute of Oncology IRCCS, Via Adamello 16, 20139, Milan, Italy
[4] Department of Oncology and Hemato-Oncology, University of Milan, Via Santa Sofia 9, 20122, Milan, Italy
[5] Computational Biology Research Centre, Human Technopole, Viale Rita Levi-Montalcini 1, 20157, Milan, Italy
[6] Department of Biology and Biotechnology "C.Darwin", Sapienza University of Rome, Rome, Italy

## MATERIALS AND METHODS

### *Curation*

Autism associated genes, as annotated by the SFARI initiative, were ranked according to their SFARI score and to the number of resources listing them for association to autism [1–5]. Starting from highly ranked genes we have looked by standard Medline searches for published information reporting the consequences of disrupting gene function on downstream proteins. The curation of the reported experimental evidence was according to the SIGNOR database curation standards [6].

### *Datasets*

We accessed SIGNOR data using available RESTAPIs in June 2022 [7]. At the time of writing SIGNOR annotates 32,200 interactions, between 8970 biological entities, 6700 of which are proteins. The SFARI genes list was downloaded from the SFARI resource in February 2021 (https://www.sfari.org/resource/sfari-gene/) [8]. Annotations as SFARI score 1 (207 proteins), 2 (211 proteins), 3 (506 proteins) and S (79 proteins) refer to the reliability of the annotation in the original resource, namely: category 1 (here indicated as SFARI score 1): High Confidence; category 2 (SFARI score 2): Strong Candidate; category 3 (SFARI score 3): Suggestive Evidence; category S (SFARI Syndromic protein). Syndromic mutations correlate with increased risk of ASD and are linked to additional characteristics not required for diagnosis.

Additional neurodevelopmental disorders protein lists were obtained from the Psychiatric Cell Map Initiative dataset [9]. 62 proteins are associated in these lists to autism spectrum disorders (PCMI-ASD), 28 to intellectual disability (PCMI-ID), 14 to epileptic encephalopathies (PCMI-EE) and 7 to schizophrenia (PCMI-SCZ).

*Network Visualization*

Direct causal connections between SFARI genes were obtained by querying by the search mode *connect* the SIGNOR database using the *SIGNOR Cytoscape App* [7]. The complete list of interactions is provided in Supplementary Table 2. Statistical significance of the tendency to form clusters is measured as a p-value calculated by comparing the number of connections made by the SFARI proteins in 1000 randomly generated networks (one-tail t-test). Network randomization is obtained using the *BiRewire* method [10] which preserves the characteristics of the nodes in the reference networks (i.e. their degrees and connection signs).

To obtain causal interaction graphs for neurodevelopmental diseases we used gene-disease association defined by the PCMI to query the SIGNOR database using the SIGNOR Cytoscape App (search mode: 'connect + add bridge proteins'). We obtain the following networks: PCMI-ASD (60 nodes; 105 edges), PCMI-ID (41 nodes; 91 edges), PCMI-EE (17 nodes; 31 edges), PCMI-SCZ (0 nodes; 0 edges). We eventually merged the three networks using Cytoscape built-in function 'merge' to obtain an interactome of 103 nodes and 209 edges.
KEGG and GO over-representation analyses (ORA) were performed using *gprofiler2* R package [11], using KEGG pathway and Gene Ontology-provided 'Biological Process' terms. In addition, we used the default background (human proteome) and filtered for Bonferroni-corrected p-value $< 0.05$ (the complete result of the ORA analysis is provided in Supplementary Table 3).

*Community detection*

To detect communities/clusters, we used the *cluster_walktrap* function within the *iGraph* R package [12].We adopted default options and in addition we used the SIGNOR score as a weight parameter, and set the maximum number of steps allowed to 4.

*ProxPath - estimating the Impact of a Protein on a phenotype.*

The *ProxPath* algorithm can estimate the regulatory impact of an input list of proteins, in our case SFARI (score 1) proteins, over phenotypes annotated in SIGNOR. Significantly-proximal phenotypes are identified using a strategy adapted from Iannuccelli et al., 2022 [13]. The pipeline consists of two steps:
STEP1: Browsing the SIGNOR interactome to create phenotype-proximity annotations. Briefly, we make use of the graph representation of the human causal network annotated in SIGNOR 3.0. For each phenotype, the algorithm retrieves all the directional paths (of length of four steps or fewer) connecting any protein in SIGNOR to the phenotype. As any step in the graph has a score (s), we define the distance between any two interacting proteins as $d = 1- s$ and the path distance score (d.s.) of a path including more than two nodes as the sum of the distances of the edges forming the path. The lower the path distance score, the shorter is the 'functional distance' and more functionally relevant is estimated the path. Next, we classify as

proteins having an influence on phenotype activity those proteins having Z-score in the path distance distribution lower than $Z = -1.96$ (i.e. -2 standard deviations, p-value <0.05) that are considered to be in a significantly small distance (close) and as having a significant chance of impacting the target phenotype. The Z-score is computed using the mean, over the distribution of path distance scores considering the distance between every protein in SIGNOR to a given phenotype. Nodes that cannot connect with four steps or fewer to phenotypes were arbitrarily assigned the highest path distance score in the distribution.
The paths connecting the query protein to a phenotype are characterized by a distance and a sign that specifies whether the protein is inferred to have a positive or a negative effect on phenotype activity. Proteins connected by paths formed by an odd number of inhibitory steps are defined as inhibitors, otherwise are considered as activators.

Identification of the paths linking a query gene to the phenotype was programmatically implemented using the *all simple_paths* function of the *NetworkX* module of the Python language [14]. The function returns every short path linking any two nodes in an oriented graph. We set a length cut-off of 4 as input parameter in order to explore only pathways with a length that is shorter or equal to the chosen threshold. R scripting was used to run python scripts and to analyse results.

STEP2: phenotype proximity enrichment of SFARI (score 1) proteins (SFARI1). To identify the proximal phenotypes impacted by the SFARI1 proteins and the resulting effect on the phenotype, we used the strategy outlined in the previous section to link 207 SFARI proteins to the up- or down-regulation of 196 phenotypes annotated in SIGNOR. We used the two-sided t-test to assess whether the proportion of paths starting from a SFARI1 protein and leading to the up-/down-regulation of a phenotype is significantly greater than the mean extracted from a randomized dataset. Briefly, we generated lists of 207 proteins that were randomly chosen among the proteins annotated in SIGNOR. 207 corresponds to the number of SFARI1 proteins. We generated 1000 of such random genes lists (The entire human proteome was considered as a background of the analysis) and, for each, we evaluated the fraction of short paths (<= 4 steps) starting from the input protein and impacting the up- or down-regulation of each phenotype displayed in SIGNOR. We eventually charted the distribution of these fractions, performed a one-tail t-test and selected phenotypes that display a Benjamini-Hochberg-corrected p-value < 0.05.

*ProxPath - estimating the Impact of a Protein on a pathway*
The *ProxPath* algorithm can estimate the regulatory impact of a list of INPUT proteins, in our case SFARI1 (i.e. SFARI, score 1) proteins, on pathways annotated in SIGNOR. Significantly-proximal pathways are identified using a strategy already described in Perfetto et al., 2021 [15]. Briefly, we define a global distance, or proximity score, between a query protein and a pathway, by considering the paths between any protein in SIGNOR and all the proteins in the pathway. To this end we followed a four-step strategy: 1) We searched the cell causal interactome for paths of four steps, or less, linking any of the query proteins and each protein in the pathway-list; 2) We select, for each query protein-pathway protein pair, the path with the shortest distance (lower distance score, d.s. - see “Estimating the Impact of a Protein on a phenotype

paragraph"); 3) If a query protein is connected to more than one protein in the pathway, we use an analogy with a parallel resistor and define the proximity score as the reciprocal of the sum of the reciprocals of the distances (d.s.) of each path linking the query protein to proteins in the pathway; 4) we classify as proteins having an influence on the pathway activity those query proteins having Z-score in the proximity score distribution lower than $Z = -1.96$ (p-value $<0.05$) The Z-score is computed over the distribution of proximity scores considering the distance between every protein in SIGNOR to a given pathway. To identify the proximal pathways impacted by the SFARI1 proteins, we used the statistical tests already described in the "Estimating the Impact of a Protein on a phenotype" paragraph.

*SIGNOR Pathway Over-representation analysis (ORA)*

To perform classical pathway over-representation analysis of SFARI1 proteins, we considered all the pathways in SIGNOR, and then we extracted all the pathway members using SIGNOR REST APIs. For each pathway, we next performed Fisher-exact test using as input the SFARI1 proteins and as background the entire human proteome. P-values were corrected for multiple testing using Benjamini-Hochberg method.

*Gene Set Enrichment Analysis of protein Proximity (GSEA)*

To benchmark the prioritization of genes based on the proximity to SFARI1 nodes we applied a gene set enrichment analysis to detect whether ASD-associated genes provided by two independent resources and methods are enriched in genes that are ranked high by our network distance approach top-close distance to SFARI1 genes. We used different databases, including the SFARI1 (as positive control), SFARI2 and 3 proteins along with two independent datasets:

- The list of Autism Spectrum disorders proteins annotated by the Psychiatric Cell Map Initiative (PCMI-ASD).
- Experimentally-characterized physical interactors of ASD proteins [16]. Recently, Pintacuda and collaborators characterized the protein-protein interaction network for 13 ASD-associated genes in human excitatory neurons derived from induced pluripotent stem cells (iPSCs). For the scope of the present analysis, we selected significant interactors (log2 FC > 0 & FDR ≤ 0.1). Next, proteins that were found to be prey of two or more ASD genes were classified as 'shared interactors', whereas those that were found to be prey of one ASD gene were classified as 'unique interactors'.

Analyses in GSEA were performed on normalized (by mean-cantered z-score) ranked distances to SFARI1 proteins generated by ProxPath, as provided in Supplementary Table 4, using the *fgseaSimple* function in the *fgsea* package in R, using nperm = 1000 [17]. Significance threshold (p-value adjusted for multiple hypothesis testing) was set to 0.05.

*Network propagation analysis via heat diffusion.*

To identify the proximal phenotypes with respect to SFARI1 genes we first performed a diffusion analysis using the *diffusion_heat* plugin in Cytoscape [18], using the entire SIGNOR network as a scaffold structure; the 207 SFARI1 proteins as input and the time parameter set to 0.1. Hence, we generated 100 random sets of 207 genes represented in the network. Given

the deterministic nature of the algorithm, to avoid redundancies we imposed two restraints: i) each set could not contain more than 25% of SFARI1 genes, and ii) each set could not intersect any other by more than 50%. Next, we iteratively measured diffusion for each gene set using *diffusion_hea*t cytoscape APIs (https://github.com/idekerlab/heat-diffusion), under Python 3.7. Time parameter was kept to 0.1. We retrieved the average diffusion score of each node, and measured the z-score of diffusion heat. To assign a significance parameter to each node diffusion score we measured the cumulative distribution function with *pnorm* in R 4.1.3 applied to z-scores. P-values were corrected for multiple testing using Benjamini-Hochberg method.

*Code availability*
Developed code is available at https://github.com/SaccoPerfettoLab/ProxPath.

SUPPLEMENTARY FIGURES

Supplementary Figure 1

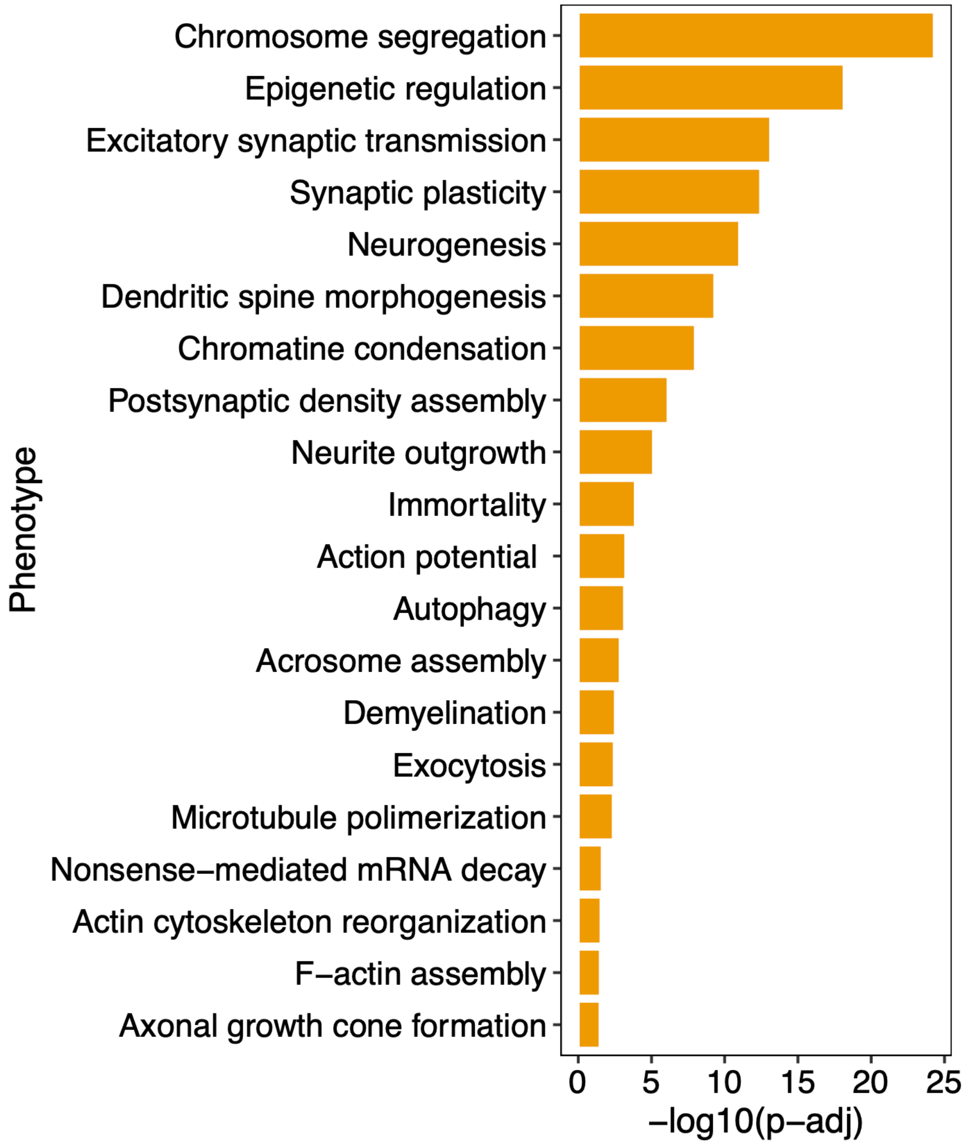


**Supplementary Figure 1. Comparison with network diffusion-based methods.** Phenotypes significantly close to SFARI1 proteins by applying network propagation algorithm, using the heat diffusion implementation provided by Carlin et al. [18]. Statistical significance was assessed using z-score, comparing the diffusion heat score of each phenotype to the mean of a randomized sample. The p-value was adjusted by applying Benjamini-Hochberg correction.

Supplementary Figure 2

A GSEA enrichment for SFARI1 proximity

| Gene List | padj | NES | Significant |
|---|---|---|---|
| PCMI ASD | 0,002 | 3,2272 | yes |
| SFARI 1 | 0,002 | 3,4123 | yes |
| SFARI 2 | 0,0613 | 1,7955 | no |
| SFARI 3 | 0,1389 | 1,4014 | no |
| Pintacuda et al, 2023 (shared interactors) | 0,002 | 2,8698 | yes |
| Pintacuda et al, 2023 (unique interactors) | 0,0496 | 1,6572 | no |
| random | 0,1187 | 1,7421 | no |

B PCMI-ASD

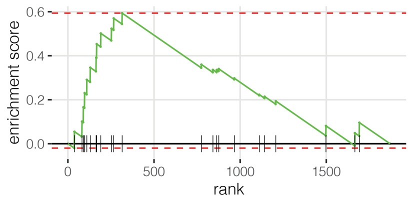


C Pintacuda et al, 2023 (shared interactors)

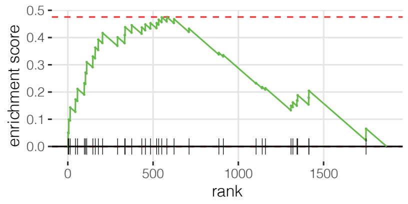


D Phenotype proximity enrichment of
Pintacuda et al, 2023 (shared interactors)

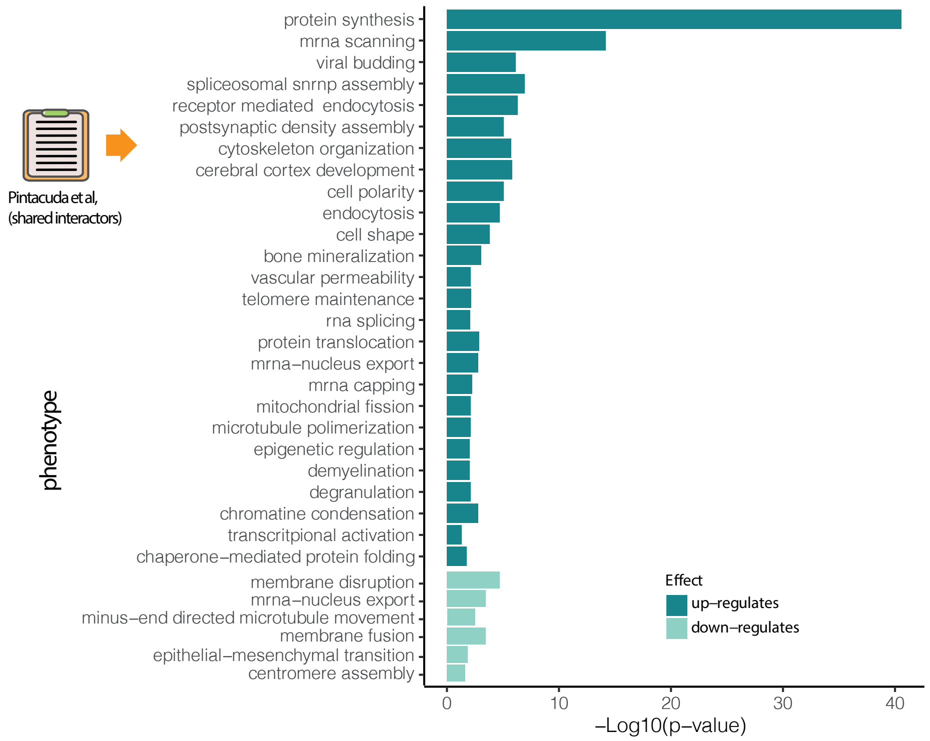


E Pathway proximity enrichment of
Pintacuda et al, 2023 (shared interactors)

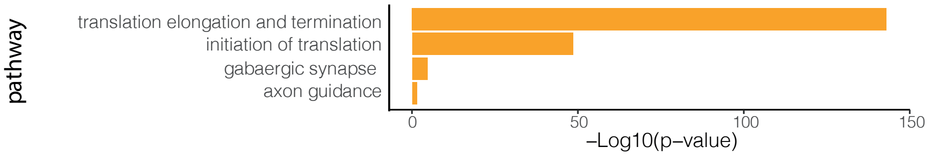


**Supplementary Figure2. Benchmarking ProxPath over independently-defined lists of ASD proteins.**

A) Gene Set Enrichment Analysis (GSEA) results where different ASD gene lists (or sets) were used to benchmark the prioritization of candidate ASD proteins based on network vicinity to SFARI1 nodes. Adjusted p-values (padj) and normalized Enrichment Scores (NES) are calculated.
B) GSEA of PCMI-ASD [9]. In green the running enrichment score for the gene set; black ticks on the abscissa indicate where the members of the gene set appear in the ranked list of proteins proximal to SFARI1 nodes. The top-ranked proximal proteins are on the left whereas on the right lay the distant proteins.
C) GSEA of the "shared interactors" of ASD proteins as defined by Pintacuda et al [16]. In green the running enrichment score for the gene set; black ticks on the abscissa show where the members of the gene set appear in the ranked list of proteins proximal to SFARI1 nodes. On the left are the top-ranked proximal proteins whereas on the right lay the most distant proteins.
D) Phenotypes that are significantly close to shared interactors of 13 ASD proteins as published by Pintacuda and collaborators. The results of the ProxPath enrichment are visualized as a bar diagram (dark and light green bars indicate up- or down-regulation of the displayed phenotype, respectively).
E) Pathways that are significantly close to shared interactors of 13 ASD proteins as published by Pintacuda and collaborators. The results of the ProxPath enrichment are visualized as a bar diagram

Supplementary Figure 3

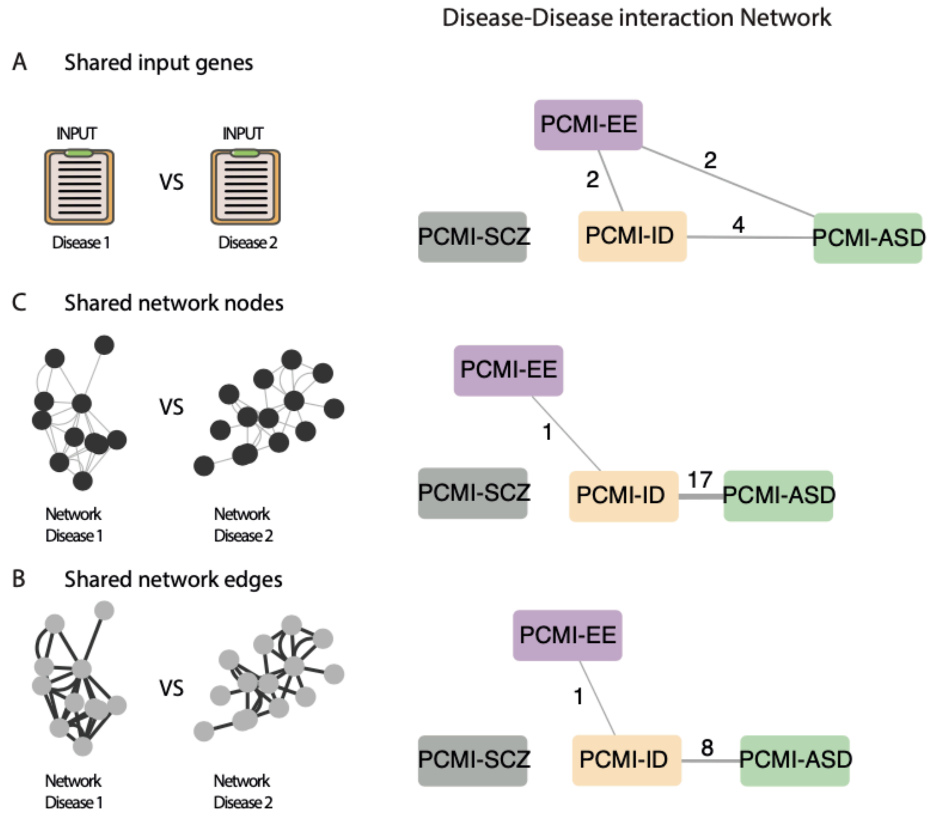


**Supplementary Figure 3. Disease-Disease Interaction Networks (DDI) of neurodevelopmental disorders.**

A) DDI where edges (and relative weights) represent the number of genes that are in common between pairs of diseases, according to the proteins annotated by the Psychiatric Cell Map Initiative (PCMI) [9] to Autism spectrum disorders (PCMI-ASD), Intellectual disability (PCMI-ID), Epileptic encephalopathies (PCMI-EE) and Schizophrenia (PCMI-SCZ) .

B) DDI where edge presence/weight reflects the number of nodes that are in common between pairs of disease Networks (as displayed in Figure 6B).

C) DDI where edge presence/weight reflects the number of edges that are in common between pairs of disease Networks (as displayed in Figure 6B).

REFERENCES


1. Pedersen CB, Bybjerg-Grauholm J, Pedersen MG, Grove J, Agerbo E, Bækvad-Hansen M, et al. The iPSYCH2012 case-cohort sample: new directions for unravelling genetic and environmental architectures of severe mental disorders. Mol Psychiatry. 2018;23:6–14.
2. Yang C, Li J, Wu Q, Yang X, Huang AY, Zhang J, et al. AutismKB 2.0: a knowledgebase for the genetic evidence of autism spectrum disorder. Database (Oxford). 2018;2018:bay106.
3. Firth HV, Richards SM, Bevan AP, Clayton S, Corpas M, Rajan D, et al. DECIPHER: Database of Chromosomal Imbalance and Phenotype in Humans Using Ensembl Resources. Am J Hum Genet. 2009;84:524–533.
4. C Yuen RK, Merico D, Bookman M, L Howe J, Thiruvahindrapuram B, Patel RV, et al. Whole genome sequencing resource identifies 18 new candidate genes for autism spectrum disorder. Nat Neurosci. 2017;20:602–611.
5. Guo H, Duyzend MH, Coe BP, Baker C, Hoekzema K, Gerdts J, et al. Genome sequencing identifies multiple deleterious variants in autism patients with more severe phenotypes. Genet Med. 2019;21:1611–1620.
6. Lo Surdo P, Iannuccelli M, Contino S, Castagnoli L, Licata L, Cesareni G, et al. SIGNOR 3.0, the SIGnaling network open resource 3.0: 2022 update. Nucleic Acids Res. 2022:gkac883.
7. De Marinis I, Lo Surdo P, Cesareni G, Perfetto L. SIGNORApp: a Cytoscape 3 application to access SIGNOR data. Bioinformatics. 2021:btab865.
8. Arpi MNT, Simpson TI. SFARI genes and where to find them; modelling Autism Spectrum Disorder specific gene expression dysregulation with RNA-seq data. Sci Rep. 2022;12:10158.
9. Willsey AJ, Morris MT, Wang S, Willsey HR, Sun N, Teerikorpi N, et al. The Psychiatric Cell Map Initiative: A Convergent Systems Biological Approach to Illuminating Key Molecular Pathways in Neuropsychiatric Disorders. Cell. 2018;174:505–520.
10. Iorio F, Bernardo-Faura M, Gobbi A, Cokelaer T, Jurman G, Saez-Rodriguez J. Efficient randomization of biological networks while preserving functional characterization of individual nodes. BMC Bioinformatics. 2016;17:542.
11. Kolberg L, Raudvere U, Kuzmin I, Vilo J, Peterson H. gprofiler2 -- an R package for gene list functional enrichment analysis and namespace conversion toolset g:Profiler. F1000Res. 2020;9:ELIXIR-709.
12. Pons P, Latapy M. Computing communities in large networks using random walks (long version). 2005.
13. Iannuccelli M, Lo Surdo P, Licata L, Castagnoli L, Cesareni G, Perfetto L. A Resource to Infer Molecular Paths Linking Cancer Mutations to Perturbation of Cell Metabolism. Front Mol Biosci. 2022;9:893256.
14. Hagberg A, Swart P, S Chult D. Exploring network structure, dynamics, and function using networkx. Los Alamos National Lab. (LANL), Los Alamos, NM (United States); 2008.
15. Perfetto L, Micarelli E, Iannuccelli M, Lo Surdo P, Giuliani G, Latini S, et al. A Resource for the Network Representation of Cell Perturbations Caused by SARS-CoV-2 Infection. Genes (Basel). 2021;12:450.
16. Pintacuda G, Hsu Y-HH, Tsafou K, Li KW, Martín JM, Riseman J, et al. Protein interaction studies in human induced neurons indicate convergent biology underlying autism spectrum disorders. Cell Genomics. 2023;3:100250.

17. Korotkevich G, Sukhov V, Budin N, Shpak B, Artyomov MN, Sergushichev A. Fast gene set enrichment analysis. 2021:060012.
18. Carlin DE, Demchak B, Pratt D, Sage E, Ideker T. Network propagation in the cytoscape cyberinfrastructure. PLoS Comput Biol. 2017;13:e1005598.
